# Identification of microRNA-Associated-ceRNA Networks Regulating Crop Milk Production in Pigeon (*Columba livia*)

**DOI:** 10.3390/genes12010039

**Published:** 2020-12-30

**Authors:** Pingzhuang Ge, Hui Ma, Yunlei Li, Aixin Ni, Adamu Mani Isa, Panlin Wang, Shixiong Bian, Lei Shi, Yunhe Zong, Yuanmei Wang, Linlin Jiang, Hailai Hagos, Jingwei Yuan, Yanyan Sun, Jilan Chen

**Affiliations:** Institute of Animal Sciences, Chinese Academy of Agricultural Sciences, Beijing 100193, China; 82101182352@caas.cn (P.G.); mahui@caas.cn (H.M.); liyunlei@caas.cn (Y.L.); 82101172347@caas.cn (A.N.); 2018y90100036@caas.cn (A.M.I.); 82101179306@caas.cn (P.W.); 82101181193@caas.cn (S.B.); 82101181189@caas.cn (L.S.); 82101185162@caas.cn (Y.Z.); 82101185166@caas.cn (Y.W.); 82101182353@caas.cn (L.J.); 2018y90100119@caas.cn (H.H.); yuanjingwei@caas.cn (J.Y.); sunyanyan@caas.cn (Y.S.)

**Keywords:** competing endogenous RNA, crop milk, lactation, pigeon

## Abstract

Pigeon belongs to altrices. Squab cannot forage independently. Nutrition can only be obtained from crop milk secreted by male and female pigeon. miRNA could regulate many biological events. However, the roles of miRNA and ceRNA in regulating crop milk production are still unknown. In this study, we investigated the miRNAs expression profile of female pigeon crop, explored the potential key genes, and found the regulatory mechanisms of crop milk production. A total of 71 miRNAs were identified differentially expressed significantly. Meanwhile, miR-20b-5p, miR-146b-5p, miR-21-5p, and miR-26b-5p were found to be the key miRNAs regulating lactation. Target genes of these miRNAs participated mainly in cell development; protein and lipid synthesis; and ion signaling processes, such as cell-cell adhesion, epithelial cell morphogenesis, calcium signaling pathway, protein digestion, and absorption. In the ceRNA network, miR-193-5p was located in the central position, and miR-193-5p/*CREBRF*/LOC110355588, miR-460b-5p/*GRHL2*/MSTRG.132954, and miR-193-5p/*PIK3CD*/LOC110355588 regulatory axes were believed to affect lactation. Collectively, our findings enriched the miRNA expression profile of pigeon and provided novel insights into the microRNA-associated-ceRNA networks regulating crop milk production in pigeon.

## 1. Introduction

Centuries ago, bird fanciers and naturalists had known that crops of parental pigeons could produce materials that nourished their springs [1]. Bernard (1859) first named it as crop milk and found that it consisted of clumps of epithelial cells shedding from the mucosal layer. As the only nutrition source of squab [2], crop milk has high nutritional value at the 1st, 2nd and 3rd weeks after hatching [3,4,5]. The lipid content of crop milk consisted mainly of triglycerides, along with phospholipids, cholesterol, free fatty acids, cholesterol esters, and diglycerides [6]. Young pigeons fed with crop milk showed remarkable growth rates, reaching 8-, 18- and 22-fold of the hatching weight. Feeding with granules including crop milk could improve the growth of domestic chickens and weaning rats significantly [7,8,9,10,11]. The crop has considerable expandability, particularly in granivores. Comparing with other poultry, except for temporary storage of ingesta and softening food, pigeon crop is more specialized for its milk production ability [12].

There were few studies on the lactating process of pigeon crop, and prolactin was the most widely investigated. As reported, prolactin promotes mitosis and early signal transduction, and growth of special epithelial cells lining the crop of pigeons could regulate the synthesis of crop milk proteins by activating the IRS1/Akt/TOR signaling pathway [13], which promotes the formation of crop milk [1,4]. Research on the molecular mechanism of pigeon crop lactation has mainly focused on the genes regulating synthesis and transport of proteins and fatty acids and their enriched pathways [14,15,16]. Gillespie has investigated the mRNA transcriptome of crop milk production and found that cornification-associated genes (including S100-A9, cornulin and A16-like) as well as β-keratin genes were up-regulated in the lactating crop. Pathway enrichment revealed the up-regulated genes involved in the proliferation of melanocytes, the adherens junction and the wingless signal pathways [17]. However, the interplay between coding and non-coding RNAs regulating the lactation process in pigeon is still unknown.

Competing endogenous RNA (ceRNA) hypothesis was proposed by Leonardo Salmena asserted [18], which states a genome-wide regulatory network could be formed through the communication of mRNA, pseudogenes, lncRNAs, and circRNAs via miRNA response elements (MREs). Experimental ceRNAs involving mRNA, pseudogenes, lncRNA, and circRNA were later verified in vivo [19]. miRNAs, therefore, remain at the center of ceRNAs cross-talk. miRNAs are about 22 nucleotides long molecules that bind to partial complementary sequences on the target RNA transcript, known as MREs, leading to inhibition or repressing of target gene expression [20,21]. The expression profile of miRNA in mammary glands of mice, cows, pigs, and other mammals was very rich [22,23,24,25], and there is lots of evidence that miRNA can regulate mammary gland development [26] and milk composition synthesis [27]. However, the research on pigeon milk production has not been carried out yet. In this study, we focused on the changes of miRNA expression between lactating and non-lactating crops and further combined the lncRNA, circRNA and mRNA data [28] to explore the potential mechanism of miRNA-associated-ceRNAs regulating crop milk production in pigeon.

## 2. Materials and Methods

### 2.1. Ethics Statement

All experiments using animals were approved by the animal care and use committee of the Institute of Animal Science, Chinese Academy of Agricultural Sciences (IAS 2018-3). All procedures were conducted in accordance with institutional animal ethics guidelines set by the Ministry of Agriculture of the People’s Republic of China.

### 2.2. Experimental Animals and Sample Collection

Ten healthy female White King pigeons were obtained from a pigeon breeding farm in Beijing, including five lactating (at lactating day 3) and five non-lactating birds. Pigeons were slaughtered by CO_2_ asphyxiation. The crop tissues were removed aseptically. Then they were cut into small pieces of 1 cm thickness and frozen in liquid nitrogen immediately.

### 2.3. RNA Isolation and Quality Assessment

The total RNA was extracted from samples of the crop tissue using TRIzol reagent (Invitrogen, Carlsbad, CA, USA) according to the manufacturer’s protocol. The RNA purity was checked using a Nanodrop Spectrophotometer (Model kaiaoK5500^®^, Beijing, China). The integrity and concentration of the RNA were measured by the RNA Nano 6000 Assay Kit of the Bioanalyzer 2100 system (Agilent Technologies, San Diego, CA, USA).

### 2.4. miRNA Library Construction and Sequencing

Total RNA was separated by 15% agarose gels to extract the small RNA (18–30 nt). After being precipitated by ethanol and centrifugal enrichment of small RNA sample, the library was prepared according to the method and process of Small RNA Sample Preparation Kit (Illumina, RS-200-0048, San Diego, CA, USA). The main contents are as follows: 1. Connecting the 3′ adaptor to the separated small RNA. 2. Connecting the 5′ adaptor to the separated small RNA. 3. Quantitative real-time PCR. 4. Recycling strips of 145–160 bp (22–30 nt RNA). After library construction, miRNA sequencing was performed by using the Illumina HiSeq^TM^ 2500 platform (Annoroad, Beijing, China) and generating 50 bp single-end reads.

### 2.5. Quality Control, Mapping and Acquisition of Sequencing Data

Raw Data were processed with perl scripts to ensure the quality of data used in the following analysis. The adopted filter criteria are: 1. Reads without 3′ adapter were filtered out. 2. Reads with the number of bases whose phred quality value was no more than 19 accounting for more than 15% were regarded as low-quality reads and were filtered out. 3. Reads with the number of N bases accounting for more than 10% were filtered out.

The reference genome (https://ftp.ncbi.nlm.nih.gov/genomes/all/GCF/000/337/935/GCF_000337935.1_Cliv_1.0/GCF_000337935.1_Cliv_1.0_genomic.fna.gz) and annotation file (https://ftp.ncbi.nlm.nih.gov/genomes/all/GCF/000/337/935/GCF_000337935.1_Cliv_1.0/GCF_000337935.1_Cliv_1.0_genomic.gff.gz) were downloaded from NCBI database. The previous mRNAs and lncRNAs sequencing data [28], which can be downloaded from China National Genomics Data Center (https://bigd.big.ac.cn/search/?dbId=gsa&q=CRA001977), were reanalyzed. These sequences were generated using libraries constructed from the same RNA used in the current study. Clean data of mRNA and lncRNA were mapped to the reference genome using HISAT2 (version 2.0.5, parameters-RNA-strandness RF-dta-t-p 4, https://daehwankimlab.github.io/hisat2/) [29]. StringTie (version 1.3.2d, parameters -G ref.gtf–rf -l, http://ccb.jhu.edu/software/stringtie/) [30] was run to build consensus sets of transcripts. As for miRNA reads, Bowtie (version 1.1.2, parameters-v0-p4-best, http://bowtie-bio.sourceforge.net/index.shtml) [31] was used to build the reference genome index and then mapthe clean reads to the pigeon genome.

### 2.6. Identification of miRNA, lncRNA and mRNA

Reads were mapped to mature miRNA and hairping, which were recorded in miRBase (release 21) [32], to identify known miRNA. Other clean reads were compared with ncRNA sequences in Rfam (version 13.0, https://rfam.org/) [33] to annotate rRNA tRNA snRNA snoRNA and other ncRNAs. After excluding the reads mapped to known miRNA, ncRNA, repeat region, and mRNA region, the remaining reads were used to predict novel miRNA by miRDeep2 (version 2.0.0.8, https://www.osc.edu/book/export/html/4389) [34].

The assembled transcripts were further subjected to a stringent filtering progress to obtain the candidate lncRNAs. First, we removed the transcripts with one exon and with lengths of less than 200 bp. Next, we calculated the reads’ coverage of transcripts using String Tie [30] and removed transcripts with a reads’ coverage of less than three. Subsequently, we eliminated known genes, pre-micro RNA and other non-coding RNAs (rRNA, tRNA, snoRNA and snRNA) using Cuffcompare.

### 2.7. Differentially Expressed Analysis of RNAs

Cuffdiff (v2.1.1) was used to calculate fragments per kilo-base of exon per million mapped fragments (FPKM) values for the lncRNAs and mRNAs, and TPM (transcripts per million) scores for the miRNAs.TPM normalization formula is as follows: TPM = (specific miRNA reads × 1,000,000)/total miRNA mapped reads. Genes with P-adj ≤ 0.05 and |log2(Fold change)|≥1 were considered as differentially expressed. DESeq (version 1.16.0, parameters-fc 2-p 0.05-q 0.05, http://www.bioconductor.org/packages/release/bioc/html/DESeq.html) [35] was used for different expression analysis.

### 2.8. miRNA Target Prediction

The miRNA targets prediction for the three other ceRNAs (mRNA, lncRNA and circRNA) was achieved using PITA (version 6, parameters-gxp-l 6-8, http://genie.weizmann.ac.il/pubs/mir07/mir07data.html) [36], miRanda (version 3.3a, parameters-sc 180-en -20-strict, http://www.microrna.org/) [37] and Target Scan (version 3.1, http://www.targetscan.org/vert_72/) [38], and at least two software intersections.

### 2.9. Functional Enrichment

The differentially expressed target genes of the differentially expressed miRNAs were subjected to Gene Ontology (GO, releases/2019-05-09, http://geneontology.org/) enrichment using hypergeometric test. Similarly, enrichment of Kyoto encyclopedia of genes and genomes (KEGG, releases/2019-05-01, http://www.kegg.jp/) of the target genes was achieved by hypergeometric test. GO and KEGG terms with *p* ≤0.05 were considered to be significantly enriched.

### 2.10. Protein-Protein Interaction Analyze

To get insight into the potential relationships of mRNAs, differentially expressed between lactating and non-lactating pigeon crops, PPI network was constructed using STRING (version 11.0, https://string-db.org/cgi/input.pl) and visualized in the cytoscape (version 3.6.1).

### 2.11. Construction of ceRNA Network

The ceRNA network was visualized using cytoscape software. The topological features of this ceRNA network were calculated by a built-in network analyzer tool in cytoscape software.

### 2.12. Quantitative Real-Time PCR

The RNA samples were reverse transcribed into cDNA using the miRcute plus miRNA first-strand cDNA Kit (TIANGEN, Beijing, China) following the manufacturers instructions. The concentration of the RNA samples was adjusted to 200 ng/μL by adding RNase free ddH_2_O. The first step of the reverse transcription was conducted at 42 °C for 60 min. It contained 5 μL RNA samples, 10 μL 2× miRNA RT reaction buffer, 2 μL miRNA RT enzyme mix, and 3 μL RNase free dH_2_O. The second step was conducted at 95 °C for 3 min. qRT-PCR was performed using the miRcute plus miRNA qPCR Kit (TIANGEN, China) and the ABI Quant Studio 7 Flex Real-Time Detection System (Life Technologies Holdings Pte Ltd., USA). Each 10 μL qRT-PCR mixture contained 5 μL 2× miRcute plus miRNA premix (SYBR&ROX), 0.2 μL reverse primer (10 μm), 0.2 μL forward primer, 1μL cDNA (100 ng), and 3.6 μL RNase free ddH_2_O. After an initial denaturing at 95 °C for 15 min, there were 40 cycles of amplification (94 °C for 20 s and 60 °C for 34 s), followed by a thermal denaturing (95 °C for 15 s, 60 °C for 60 s, and 95 °C for 15 s) to generate melting curves. The miRNA primer design software was provided by Vazyme Biotech Co., Ltd. Nanjing, China.

The RNA samples were reverse transcribed into cDNA using the PrimeScript RT Reagent Kit (TaKaRa, Dalian, China) following the manufacturer’s instructions. The concentration of the RNA samples was adjusted to 200 ng/μL by adding RNase free ddH_2_O. The first step of the reverse transcription was conducted at 42 °C for 2 min. It contained 5 μL RNA samples, 1 μL gDNA Eraser, 2 μL 5× gDNA Eraser Buffer, and 2 μL RNase free ddH_2_O. The second step was conducted at 37 °C for 15 min, then at 85 °C for 5s. It contained 10 μL last step reaction solution, 1 μL PrimeScript RT Enzyme Mix, 4 μL 5× PrimeScript Buffer, 1 μL RT Primer Mix, and 4 μL RNase free ddH_2_O. A qRT-PCR was performed using the PrimeScript One Step RT-PCR Kit (TaKaRa, Dalian, China) and the ABI Quant Studio 7 Flex Real-Time Detection System (Life Technologies Holdings Pte Ltd., Carlsbad, USA). Each 10 μL qRT-PCR mixture contained 5 μL SYBR Premix Ex TaqTM II, 0.5 μL (10 pM), and in each primer there was 0.2 μL ROX Reference Dye II (50×), 1.5 μL cDNA (100 ng) and 2.3 μL RNase free ddH_2_O. After an initial denaturing at 95 °C for 3 min, there were 40 cycles of amplification (95 °C for 30 s and 60 °C for 34 s), followed by a thermal denaturing (95 °C for 15 s, 60 °C for 60 s, and 95 °C for 15 s) to generate melting curves. The primers of the genes were designed by the Primer Premier 5.

The primers in this study were summarized in Table S1. All mRNA expression data were normalized to hypoxanthine phosphoribosyltransferase 1 (*HPRT1*). All miRNA expression data were normalized to U6. The relative expression of the genes was calculated using the 2^−ΔΔCt^ method.

### 2.13. Data Accessibility

The sequencing data have been submitted to the SRA database (accession number: PRJNA612642). SRA records could be accessed via the following link (https://www.ncbi.nlm.nih.gov/sra/PRJNA612642).

## 3. Results

### 3.1. Identification of Pigeon Small RNAs

**Figure 1:**
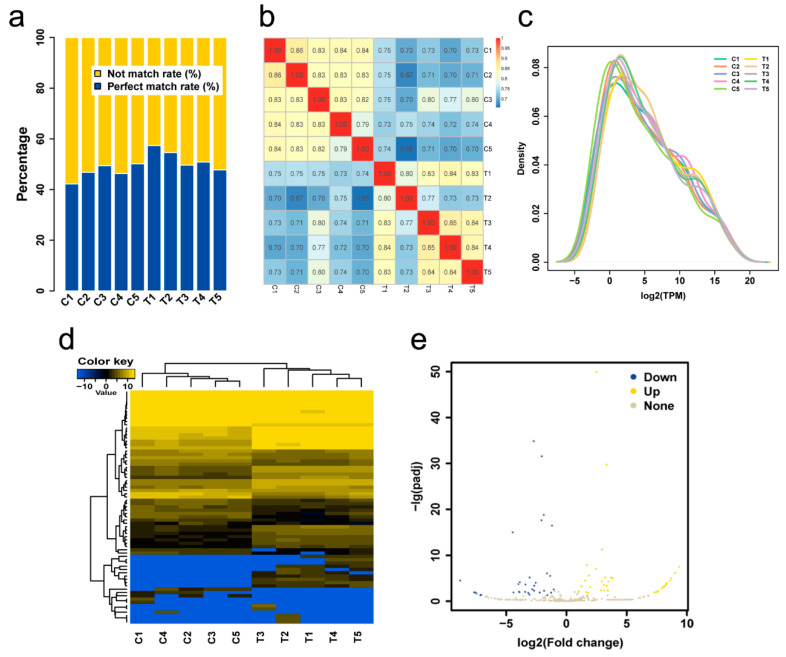
(**a**) Proportion of clean reads aligned to the pigeon reference genome. The abscissa represents the sample; the ordinate represents the percentage. (**b**) A Pearson correlation coefficients graph of individuals based on the TPM value. (**c**) Expression quantity distribution density diagram. (**d**) Heat map of differentially expressed miRNAs. (**e**) Volcano map of miRNA expression levels. Gray dots, yellow dots and blue dots represent miRNAs with no significant differences, up-regulated miRNAs and down-regulated miRNAs respectively.

As reported in Ma et al., the clean reads rate in individual samples was above 88%, and the clean Q30 bases rate was above 90% after data filtering [28]. For mapped reads, small RNAs, a total of 42.18%, 46.76%, 49.43%, 46.29%, 50.17%, 57.37%, 54.57%, 49.61%, 50.83%, and 47.74% reads from C1, C2, C3, C4, C5, T1, T2, T3, T4, and T5 were mapped to the pigeon reference genome (Table 1, Figure 1a), respectively. Known miRNA, rRNA, tRNA, snRNA, snoRNA, and novel miRNAs were identified after annotating for small RNAs. The number of known miRNAs accounted for more than 24% in all annotated small RNAs (Supplementary Table S2). In order of scale, miRNA > tRNA > rRNA > snoRNA in all the libraries sequenced. Others were less than 1%.

A total of 386 miRNAs (188 known miRNAs, 198 novel miRNAs) was found to be expressed in the crop of lactating and non-lactating pigeons (Supplementary Tables S3 and S4). A good correlation among individuals in the stage was observed in the miRNA cluster map and density distribution map (Supplementary Table S4, Figure 1b,c). A total of 50 known miRNAs and 21 novel miRNAs were differentially expressed (Supplementary Table S5, Figure 1d,e). Top 10 differential expressed miRNAs were shown in Table 2, including four up-regulated and six down-regulated ones.

### 3.2. miRNA Target Prediction and Analyze

**Figure 2:**
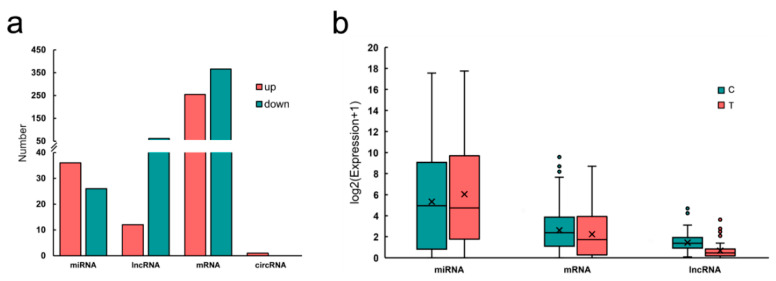
(**a**) The number of up/down regulation of different type RNAs in pigeon crop. (**b**) Cumulative distribution of RNA expression.

mRNAs and lncRNAs targets of the differentially expressed miRNA were predicted using miRanda, PITA and Target scan databases. We identified 705 pairs of miRNA-mRNA (including 39 known miRNAs, 17 novel miRNAs and 620 mRNAs), 172 pairs of miRNA-lncRNA (including 28 known miRNAs, 12 novel miRNAs and 75 lncRNAs) and a pair of miRNA-circRNA. The characteristic descriptions of RNAs are shown in Figure 2.

**Figure 3:**
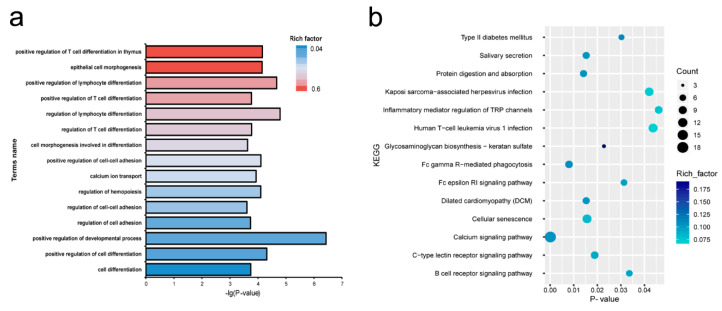
(**a**) Enriched biological process for target genes of differentially expressed miRNAs genes between lactating and non-lactating pigeon crop. (**b**) KEGG pathways enriched in target genes of the differentially expressed miRNAs between lactating and non-lactating pigeon crops.

GO and KEGG pathway analyses of the target mRNAs of differentially expressed miRNA were performed. The target genes were enriched in 52 GO biological process items and 14 KEGG pathways (Supplementary Tables S6 and S7), including development process, cell-cell adhesion, epithelial cell morphogenesis, calcium signaling pathway, protein digestion and absorption, and glycosaminoglycan biosynthesis—of keratan sulfate (Figure 3).

### 3.3. PPI Network

**Figure 4:**
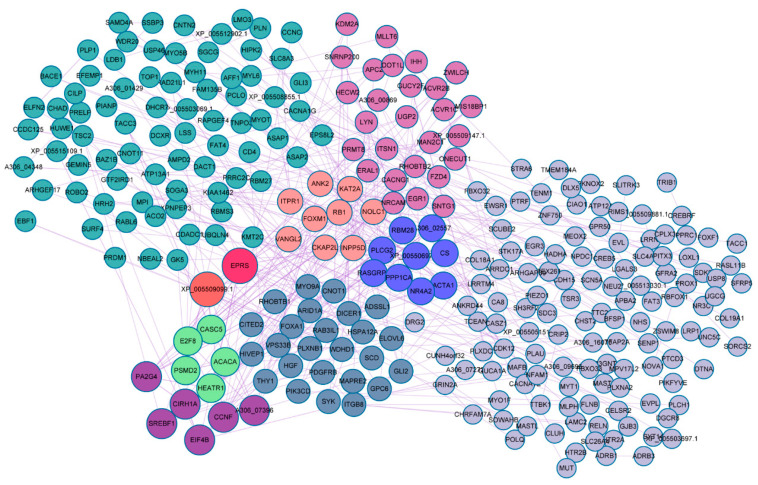
Summary of protein-protein interaction network of target genes of the differentially expressed miRNAs between lactating and non-lactating pigeon crops. Different colors represent modules, and size of the node represents connectedness of the node.

The 620 target mRNAs of the differentially expressed miRNAs were submitted to string online tool where PPI network was constructed. Over 45% of the target mRNAs were nodes in the complex PPI network (Figure 4, Supplementary Table S8). Glutamyl-prolyl tRNA synthetase (*EPRS*), XP 005509099.1, sterol regulatory element-binding protein 1 (*SREBF1*), acetyl-CoA carboxylase α (*ACACA*), and protein phosphatase type 1 α (*PPP1CA*) were identified as the most highly connected nodes in the network.

### 3.4. miRNA-mRNA-lncRNA/circRNA Interaction Network

**Figure 5:**
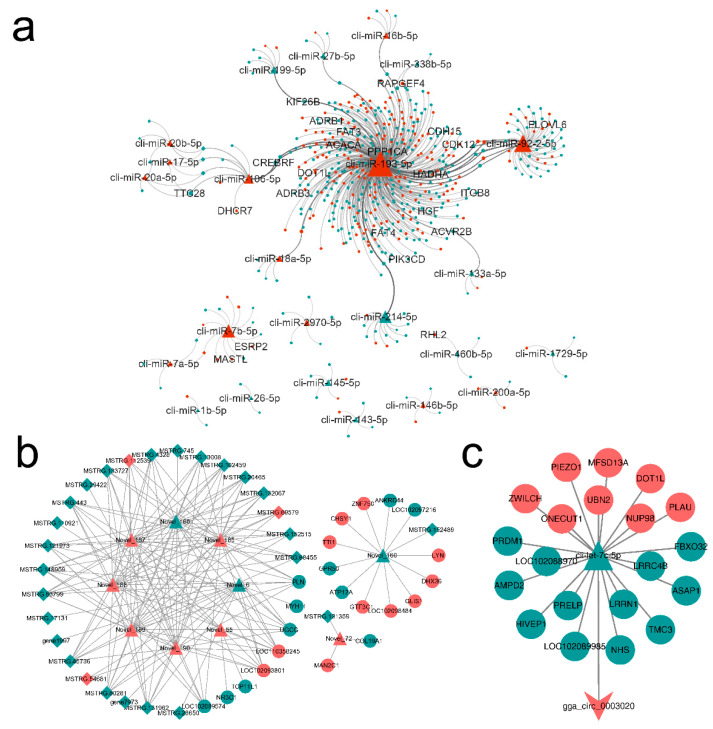
ceRNA network for target RNA molecules of the differentially expressed miRNAs between lactating and non-lactating pigeon crops. Triangular, ellipse, diamond, and V nodes represent miRNA, mRNA, lncRNA and circRNA respectively. Red represents up-regulation and green represents down-regulation. The ceRNAs were connected by edges. (**a**) known miRNA-mRNA-lncRNA network. (**b**) Novel miRNA-mRNA-lncRNA network. (**c**) miRNA-mRNA-circRNA network.

The interaction information of miRNA-mRNA, miRNA-lncRNA and miRNA-circRNA were predicted by PITA, miRanda and target scan. Based on these data, we constructed miRNA-mRNA-lncRNA/circRNA ceRNA regulatory networks and visualized them via cytoscape software. LncRNAs, circRNAs and mRNAs were connected by the same target miRNAs, and 1619 regulatory triplets (1024 pairs known miRNA-mRNA-lncRNA, 575 pairs novel miRNA-mRNA-lncRNA and 20 pairs miRNA-mRNA-circRNA) were obtained (Figure 5a–c). The topological features of ceRNA network were assessed by a built-in network analyzer tool in cytoscape software, including betweenness, network degree, and closeness centrality.

Statistics of four types of RNA degree value, betweenness centrality and closeness centrality in the network, retain higher degree value RNAs (Table 3) as the hub of ceRNA network. miR-193-5p, miR-92-2-5p, miR-7b-5p, and CREB3 regulatory factor (*CREBRF*) were at the core of the network, with the most lncRNAs and mRNAs associated with them. cli-let-7c-5p was the only miRNA connected to the lone target circRNA (circ_0003020).

### 3.5. Functional Annotation of the mRNAs in ceRNA Network

**Figure 6:**
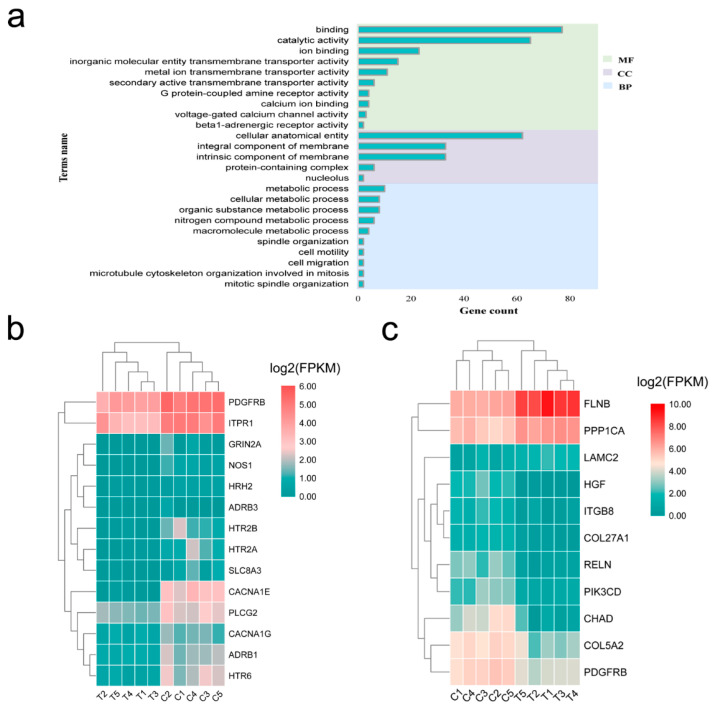
Functional enrichment analysis of the mRNA targets of the differentially expressed miRNAs between the lactating and non-lactating pigeon crops. (**a**) GO analysis; (**b**) Heatmap displaying the lactation-related genes identified in calcium signaling pathway; (**c**) Heatmap displaying the lactation-related genes identified in focal adhesion pathway. C, non-lactating group; T, lactating group.

Functional enrichment analysis of mRNA targets of differentially expressed miRNAs was conducted to explore the function of mRNA in ceRNA network. These mRNAs were significantly enriched in 25 GO terms, including cell motility, cell migration, and calcium ion binding (Figure 6a). Only calcium signaling pathway and focal adhesion were significantly enriched in the KEGG pathway. The DEGs in the pathway are shown in the heat maps (Figure 6b,c).

### 3.6. Construct Fatty Acid Biosynthesis Pathway Function Network

**Figure 7:**
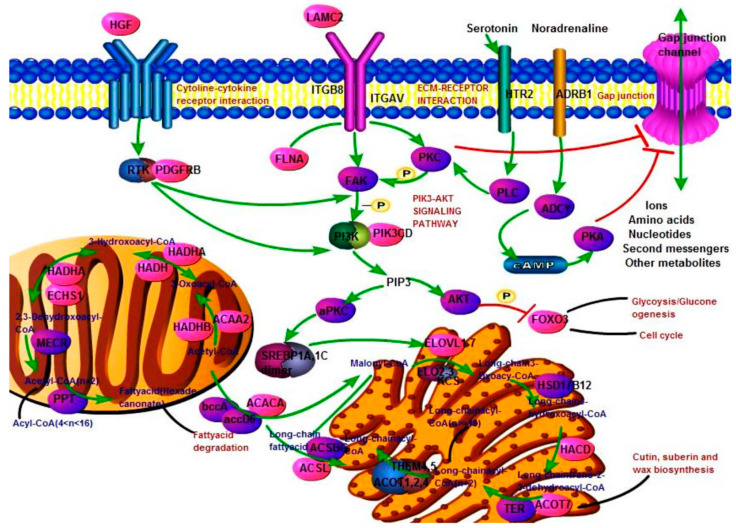
Fatty acid biosynthesis function network. The red molecule and other color molecule respectively represent DEGs and unidentified genes. The green line and the black line represent direct connection and indirect connection. The blue and red letters represent chemical molecules and pathways.

Lipids were extremely important nutrients in crop milk, and we identified many genes that played an important role in the extension, synthesis and metabolism of fatty acids. So, we constructed a metabolic sub-network related to fatty acid biosynthesis (Figure 7). Metabolic networks were involved in insulin, PI3K-Akt, ECM-receptor interaction, GAP junction, fatty acid biosynthesis and extension, FoxO, and cell cycle signaling pathways. Purple ovals represent proteins involved in pathways. Pink ovals represent the differentially expressed genes identified in this study. The nine genes (hepatocyte growth factor (*HGF*), laminin subunit γ 2 (*LAMC2*), integrin subunit β 8 (*ITGB8*), platelet derived growth factor receptor β (*PDGFRB*), phosphatidylinositol-4,5-bisphosphate 3-kinase catalytic subunit delta (*PIK3CD*), adrenoceptor β 1 (*ADRB1*), *ACACA*, ELOVL fatty acid elongase 6 (*ELOVL6*), hydroxyacyl-CoA dehydrogenase/3-ketoacyl-CoA thiolase/enoyl-CoA hydratase (trifunctional protein), and α subunit (*HADHA*) were involved in the ceRNA network.

As shown in Figure 7, these differentially expressed genes were involved in various steps of fatty acid elongation and synthesis. In particular, *ACACA* and elongation of very long-chain fatty acid proteins (*ELOVL*) have also been identified as differentially expressed. The synthesized products were also associated with signal pathways such as cutin, suberin and wax biosynthesis and fatty acid degradation.

### 3.7. The Exploration of ceRNA Regulates Axes

The key regulation axis of ceRNA regulating the crop milk production was screened by integrating the results of targeted relationship and functional regulation network. The results are shown in Table 4. miR-193-5p and many associated with pigeon milk production key genes were predicted as a target relationship. MSTRG.65211 and LOC110355588 might be in competition with miR-193-5p to regulate gene expression.

### 3.8. Validation of Differentially Expressed RNAs

**Figure 8:**
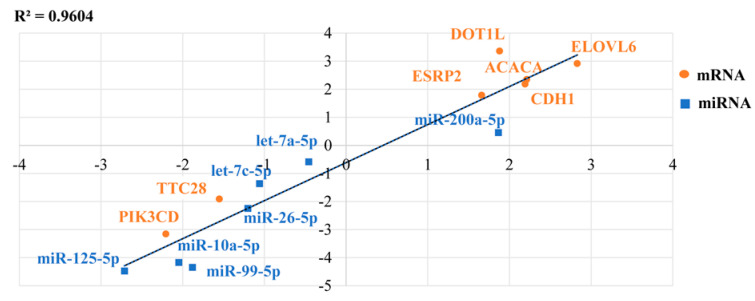
An illustration of the qRT-PCR confirmation for the RNA-seq. The correlations of the gene expression level of the 14 differentially expressed genes between lactating and non-lactating crops using RNA-Seq and a qRT-PCR. The X-axis and Y-axis show the log_2_Fold change (T/C) measured by the RNA-seq and qRT-PCR, respectively. T represents lactating group and C represents non-lactating group. The hypoxanthine phosphoribosyltransferase 1 (*HPRT1*) gene was used as a housekeeping internal control.

A total of seven miRNAs and seven mRNAs were chosen for qRT-PCR validation of the RNA sequencing results in lactating and non-lactating pigeon crop. *ACACA*, E-cadherin (*CDH1*), *ELOVL6*, DOT1 like histone lysine methyltransferase (*DOT1L*), epithelial splicing regulatory protein 2 (*ESRP2*), and miR-200a-5p were identified to be upregulated in lactating crop compared to non-lactating, whereas tetratricopeptide repeat domain 28 (*TTC28*), *PIK3CD*, let-7c-5p, let-7a-5p, miR-26-5p, miR-10a-5p, miR-99-5, and miR-125-5p were downregulated (Figure 8). These results were consistent with the sequencing results. Therefore, differential expression of these mRNAs might be involved in the process of crop milk production.

## 4. Discussion

Male and female pigeons have the ability to produce nutrients in their crop for the nourishment of their young. The production of the nutrient has been likened to lactation in mammals, and hence, it has been called crop milk [39]; meanwhile, the pigeon crop undergoes significant changes to the tissue structure during lactation [17]. miRNAs have demonstrated to play crucial functions in lipid metabolism, adipose tissue development and immune response [40,41,42]. In the present study, we found that over 40% of the small RNAs in pigeon crop were miRNAs; a total of 71 miRNAs were identified as significantly differentially expressed. We hypothesized that these differentially expressed miRNAs are involved in crop tissue morphological changes and pigeon milk synthesis. Histological studies showed that the layers of cells in the crop during lactation proliferated and that there were shed epithelial cells in the pigeon milk [43,44]. We noticed that many miRNAs involved in cell proliferation and migration have been identified, such as miR-20b-5p, which was proved to participate in the epithelial mesenchymal transition (EMT), migration and invasion process of prostate cancer [45]. MiR-146b-5p played a regulatory role in the proliferation and differentiation of human fibroblasts and chicken myoblasts [46,47]. MiR-21-5p is up-regulated in a variety of cancer cells such as non-small cell lung cancer, Hepatocellular carcinoma, melanoma, and colon adenocarcinoma cells, and has been shown to promote cell proliferation [48,49,50,51]. MiR-193-5p downregulation has significant angiogenic effect by inducing migration and proliferation in myocardial microvascular endothelial cells. Insulin-like growth factor 2 (IGF2) acted as a direct regulator to prevent this process from happening [52]. Gillespie suggested that the exfoliation of the crop epithelium due to inadequate blood supply leads to cellular keratinization [39], which may be an effect of miR-193-5p upregulation and inhibition of angiogenesis during lactation. Furthermore, Luo et al. found that miR-26b-5p serves as a direct negative regulator of TCF-4 expression within human adipose-derived mesenchymal stem cell, leading to inactivation of the Wnt/β-catenin pathway and thereby promoting the adipogenic differentiation of these cells in vitro [53]. This function is closely related to the characteristics of pigeon milk with high fat and nutrition. Based on the above evidence, we speculated that these miRNAs played an important role in the proliferation and exfoliation of the crop epithelial cells during the lactation period.

In order to further explore the functions of miRNAs, target gene prediction and functional enrichment analysis were performed. We identified 620 differentially expressed target genes and 75 differentially expressed target lncRNAs for differentially expressed miRNAs. By performing functional enrichment analysis of these genes, it was found that GO categories and pathways for DEGs such as development process, cell-cell adhesion, epithelial cell morphogenesis, calcium signaling pathway, protein digestion and absorption, and glycosaminoglycan biosynthesis of keratan sulfate signaling pathways were enriched between lactation and non-lactation periods. PPI analysis revealed *EPRS*, *SREBF1* and *ACACA* closely associated with adipogenesis, degeneration, and lipid accumulation [54,55]. These results suggested that genes regulating substance synthesis remained active to support the synthesis of a large number of milk compositions in lactation.

Further, we found several GO terms related to cell adhesion and calcium signaling pathway. Classical cadherins are the key molecules that control cell-cell adhesion and are essential regulators of tissue homeostasis that govern cellular function and development, by transducing adhesive signals to a complex network of signaling effectors and transcriptional programs [56]. Specifically, *CDH1* and M-cadherin (*CDH15*) are calcium-dependent intercellular adhesion molecules. *CDH1* played an important role in epithelial adhesion, geometrical packing and Spatial effort [57,58]. We suspect *CDH15* has a similar function; Claudins as the cell adhesion protein are members of the membrane-associated guanylate kinases (MAGUK) family proteins and the main bridge proteins of connecting claudins with the underlying actin cytoskeleton [59]. Studies found that the formation of adhesion junctions could also promote tight junction assembly by altering the lipid composition of the plasma membrane. Low Ca^2+^ led to internalization of claudins and reduced cholesterol in the plasma membrane [60]. This suggested that Ca^2+^ concentration and the genes involved in cell adhesion might be related to the shedding of epithelial cells in the crop of lactating pigeon. Other genes involved in cell adhesion played a major role in the immune system and neural system, majorly participating in T cell receptor signaling pathway, inducing T cell activation and differentiation [61].

Regarding the constructed miRNA-associated-ceRNA networks, we desired to explore the roles of key RNA molecules in crop milk formation and epithelial cell morphogenesis. We found that many genes were identified as predicted target genes of miRNAs that might play critical roles in metabolism through several pathways. So *HADHA*, adrenoceptor β 3 (*ADRB3*), *ACACA*, *ELOVL6,* and 7-dehydrocholesterol reductase (*DHCR7*) were identified as key genes for lipid and fatty acid metabolism. The product of *ACACA* is the rate-limiting enzyme in the biosynthesis of long-chain fatty acids and was reported to be an important enzyme involved in the synthesis of saturated fatty acids. This enzyme was expressed ubiquitously, but the highest level was found in lipogenic tissues such as the liver, adipose tissue and lactating mammary gland [62,63,64]. Similarly, *ADRB3* was a key gene in the process of adipose tissue browning and was reportedly down-regulated in adipocytes of obese people [65]. This allowed researchers to focus on adipose tissue browning in mice [66] and humans [67,68]. In this study, *ADRB3* was downregulated in lactating pigeon crop, corresponding to the period of a high proportion of fat (33.8%) in crop milk [4]. Both *HADHA* and *DHCR7* have been shown to be involved in lipid processes, including cardiolipin re-modeling, fatty acid β-Oxidation in human myocardium [69] and vitamin D-lipid interaction [70], respectively. Seven members of the ELOVL protein family have been described in mammals [71,72]. They are closely related to fatty acid metabolism and hepatic insulin sensitivity. ELOVL fatty acid elongase 4 (*ELOVL4*), ELOVL fatty acid elongase 5 (*ELOVL5*), *ELOVL6,* and ELOVL fatty acid elongase 7 (*ELOVL7*) were differentially expressed and identified in the crop tissues of lactating and non-lactating pigeons in our previous study [28]. However, only *ELOVL6* appeared in the ceRNA network in the current study. *ELOVL4* could be capable of elongating both saturated fatty acids and polyunsaturated fatty acid (PUFA) [71] and was responsible for the biosynthesis of saturated very long-chain fatty acids (VLC-FA) that are components of sphingolipids and ceramides and are important in the skin [73,74]. *ELOVL6* and *ELOVL7* can catalyze the elongation of saturated and monounsaturated fatty acids in mammals. *ELOVL6* deficiency in mouse livers changes the composition of liver fatty acids, the length of the fatty acid chain, and the ratio of fatty acids, with a consequent reduction of SREBP-1 and PPARα in the liver. Decreased expression of *SREBP-1* reduced fatty acid synthesis, but increased insulin sensitivity [75]. In this study, *ELOVL6* was up-regulated in the crop of lactating pigeon, suggesting that it may be involved in fat synthesis.

It has been established that crop milk was formed mainly from proliferation and shedding of a large number of epithelial cells in the crop tissue. Indian Hedgehog (*IHH*) could regulate formation and proliferation of mesenchymal cells, which in turn affect epithelial proliferation and differentiation in intestinal epithelial cell [76]. Grainy head-like transcription factor 2 (*GRHL2*) was a member of grainy head-like transcription family, which played a fundamental role in epidermal integrity, embryonic neural tube closure, and wound healing processes [77,78]. *ESRP2* protein was reported to regulate alternative splicing in epithelial cells [79,80]. Its loss disrupted the splicing of the transcripts from genes, thereby losing the ability to form sheets of cells with junctions between them [81]. In addition, *ESRP2* and microtubule-associated serine/threonine kinase like (*MASTL*) clustered with epithelial markers and correlated inversely with the expression of mesenchymal markers [79,81]. EMT could activate a molecular program that drives epithelial cells to acquire mesenchymal phenotypes; it usually ends with the transition from mesenchymal to epithelial (MET). This state transition was very flexible [82]. Interestingly, this seems to be similar to the transformation between lactating and non-lactating crop tissues, and we infer that the *ESRP2* gene was transformed between lactating and non-lactating crop tissues during the two periods. More importantly, in many cancers, the property of tumor dissemination and metastasis is closely associated with re-enabling developmental properties such as EMT [83]. These results indicated that the formation of crop milk is related to the transformation of cell morphology. Kinesin family member 26B (*KIF26B*) stabilized the cell-cell adhesion of mesenchymal cells in the developing kidney through enhancing the interaction of NMHC II and actin [84]. *DOT1L* between renal fibroblast activation and epithelial mesenchymal played a role in the process of transformation [85]. Phosphatidylinositol-3-kinase (PI3K) was a key signaling hub in immune cells. Either too little or too much PI3K activity was deleterious [86]. Mutations, activation, and low expression of *PIK3CD* were associated with autoimmune diseases and immunodeficiency [87,88].

In summary, a regulatory network was constructed. The regulatory relationship of these key genes has been listed and could be used as a key research object to further explain the production mechanism of crop milk. However, a limitation of this study is that most of the ceRNA networks identified were largely based on correlation analysis. Further strong evidence should be obtained to fully elucidate the identified ceRNA regulatory networks. Collectively, our study offered new insight for the ceRNA regulatory in crop milk production.

## 5. Conclusions

MiRNAs were abundant in the pigeon crop. Substance synthesis and cell morphogenesis genes remained active in lactation under the miRNA-associated-ceRNA regulation. Our findings enriched the pigeon miRNA expression profile and provide novel insights into the miRNA-associated ceRNA expression pattern and regulatory roles in crop milk production.

## Figures and Tables

**Table 1:** Basic and mapping data of small RNA sequencing.

Sample	Total Reads	Match Reads	Match Rate, %	Not Match Rate, %
C1	23,186,308	9,780,560	42.18	57.82
C2	24,761,407	11,578,750	46.76	53.24
C3	21,598,417	10,675,774	49.43	50.57
C4	20,696,077	9,580,916	46.29	53.71
C5	25,248,936	12,668,115	50.17	49.83
T1	21,729,292	12,465,242	57.37	42.63
T2	21,760,177	11,875,079	54.57	45.43
T3	30,614,359	15,187,405	49.61	50.39
T4	32,883,572	16,714,329	50.83	49.17
T5	31,975,781	15,266,339	47.74	52.26

**Table 2:** Top 10 differentially expression miRNAs.

Name	Log_2_ (FC)	*p*-Value	Label
cli-miR-7a-5p	3.32	1.85 × 10^−32^	up
cli-miR-20b-5p	2.97	1.29 × 10^−13^	up
cli-miR-21-5p	2.49	2.86 × 10^−53^	up
cli-miR-146b-5p	1.72	3.66 × 10^−10^	up
cli-miR-26-5p	−1.20	6.57 × 10^−19^	down
cli-miR-99-5p	−1.88	2.08 × 10^−21^	down
cli-miR-10a-5p	−2.05	2.14 × 10^−34^	down
cli-miR-100-5p	−2.08	3.72 × 10^−20^	down
cli-miR-125-5p	−2.71	7.37 × 10^−38^	down
cli-miR-145-5p	−4.45	2.01 × 10^−17^	down

**Table 3:** Hub RNAs of ceRNA network.

Name	Betweenness Centrality	Closeness Centrality	Degree	Class
cli-miR-193-5p	0.99	0.70	389	miRNA
cli-miR-92-2-5p	0.18	0.33	53	miRNA
cli-miR-7b-5p	0.93	0.77	14	miRNA
cli-miR-214-5p	0.05	0.30	13	miRNA
cli-miR-106-5p	0.06	0.30	10	miRNA
Novel_189	0.09	0.58	22	miRNA
Novel_188	0.09	0.58	22	miRNA
Novel_187	0.09	0.58	22	miRNA
cli-let-7c-5p	1.00	1.00	21	miRNA
TTC28	0.01	0.23	3	mRNA
CREBRF	0.03	0.42	2	mRNA
SCML4	0.03	0.42	2	mRNA
RAPGEF4	0.00	0.41	2	mRNA
STRA6	0.02	0.45	2	mRNA
LOC110358245	0.13	0.60	8	mRNA
PLN	0.02	0.50	7	mRNA
UGCG	0.02	0.50	7	mRNA
DOT1L	0.00	0.51	1	mRNA
MSTRG.71575	0.01	0.23	3	lncRNA
MSTRG.151245	0.01	0.23	3	lncRNA
MSTRG.2421	0.01	0.23	3	lncRNA
LOC110360772	0.00	0.16	2	lncRNA
MSTRG.81107	0.14	0.53	2	lncRNA
MSTRG.1547	0.14	0.53	2	lncRNA
circ_0003020	0.00	0.51	1	circRNA

**Table 4:** Key ceRNA regulation axis.

miRNA	Label	mRNA	Label	lncRNA	Label
cli-miR-193-5p	up	CREBRF	down	MSTRG.65211	down
cli-miR-193-5p	up	ADRB1	down	MSTRG.65211	down
cli-miR-193-5p	up	IHH	down	MSTRG.65211	down
cli-miR-193-5p	up	ADRB3	down	MSTRG.65211	down
cli-miR-193-5p	up	CDH15	down	MSTRG.65211	down
cli-miR-193-5p	up	PIK3CD	down	MSTRG.65211	down
cli-miR-193-5p	up	ITGB8	down	MSTRG.65211	down
cli-miR-193-5p	up	HGF	down	MSTRG.65211	down
cli-miR-193-5p	up	PDGFRB	down	MSTRG.65211	down
cli-miR-193-5p	up	CREBRF	down	LOC110355588	down
cli-miR-193-5p	up	ADRB1	down	LOC110355588	down
cli-miR-193-5p	up	ADRB3	down	LOC110355588	down
cli-miR-193-5p	up	CDH15	down	LOC110355588	down
cli-miR-193-5p	up	PIK3CD	down	LOC110355588	down
cli-miR-193-5p	up	ITGB8	down	LOC110355588	down
cli-miR-193-5p	up	HGF	down	LOC110355588	down
cli-miR-193-5p	up	PDGFRB	down	LOC110355588	down
cli-miR-193-5p	up	IHH	down	LOC110355588	down
cli-miR-106-5p	up	CREBRF	down	MSTRG.71575	down
cli-miR-106-5p	up	CREBRF	down	MSTRG.2421	down
cli-miR-106-5p	up	CREBRF	down	MSTRG.151245	down
cli-miR-338b-5p	down	RAPGEF4	up	MSTRG.20439	down
cli-miR-460b-5p	down	GRHL2	up	MSTRG.132954	down
cli-let-7c-5p	down	DOT1L	up	circ-0003020	down

## Supplementary Materials

Supplementary materials can be found at https://www.mdpi.com/2073-4425/12/1/39/s1. Table S1: Primers of lncRNAs and mRNAs used in qRT-PCR. Table S2: The annotation file of Small RNA. Table S3: The information of Novel RNA. Table S4: The TPM value of miRNA. Table S5: The differential expression of miRNA. Table S6: The GO terms of enriched. Table S7: The KEGG pathway of enriched. Table S8: The information of protein interactions.

## Abbreviations

*MREs*	miRNA response elements
GO	Gene ontology
*KEGG*	Kyoto encyclopedia of genes and genomes
ceRNA	Competing endogenous RNA
*HPRT1*	Hypoxanthine phosphoribosyltransferase 1
*DEGs*	differentially expressed genes
*PPI*	Protein protein interaction
*EPRS*	Glutamyl-prolyl tRNA synthetase
*SREBF1*	Sterol regulatory element-binding protein 1
*ACACA*	Acetyl-CoA carboxylase α
*PPP1CA*	Protein phosphatase type 1 α
*CREBRF*	CREB3 regulatory factor
*ELOVL4*	ELOVL fatty acid elongase 4
*ELOVL5*	ELOVL fatty acid elongase 5
*ELOVL6*	ELOVL fatty acid elongase 6
*ELOVL7*	ELOVL fatty acid elongase 7
*HGF*	Hepatocyte growth factor
*LAMC2*	Laminin subunit γ 2
*ITGB8*	Integrin subunit β 8
*PDGFRB*	Platelet derived growth factor receptor β
*ADRB1*	Adrenoceptor β 1
*PIK3CD*	Phosphatidylinositol-4,5-bisphosphate 3-kinase catalytic subunit delta
*CDH1*	E-cadherin
*DOT1L*	DOT1 like histone lysine methyltransferase
*ESRP2*	Epithelial splicing regulatory protein 2
*TTC28*	Tetratricopeptide repeat domain 28
*IGF2*	Insulin-like growth factor 2
*CDH15*	M-cadherin
*MAGUK*	Membrane associated guanylate kinases
*ADRB3*	Adrenoceptor β 3
*DHCR7*	7-dehydrocholesterol reductase
*IHH*	Indian Hedgehog Regulates
*GRHL2*	Grainy head like transcription factor 2
EMT	Epithelial mesenchymal transition
MET	Mesenchymal epithelial transition
*KIF26B*	Kinesin family member 26B
PUFA	polyunsaturated fatty acid
VLC-FA	very long-chain fatty acids
